# Takotsubo syndrome in a cancer patient treated with a combination of anti-cancer drugs including immune checkpoint inhibitors: a case report

**DOI:** 10.1093/ehjcr/ytae355

**Published:** 2024-07-23

**Authors:** Keita Yamada, Mizuki Ida-Ichikawa, Naoki Fujimoto, Masaki Ishida, Kaoru Dohi

**Affiliations:** Department of Cardiology and Nephrology, Mie University Graduate School of Medicine, 2-174 Edobashi, Tsu, Mie 5148507, Japan; Department of Cardiology and Nephrology, Mie University Graduate School of Medicine, 2-174 Edobashi, Tsu, Mie 5148507, Japan; Department of Cardiology and Nephrology, Mie University Graduate School of Medicine, 2-174 Edobashi, Tsu, Mie 5148507, Japan; Department of Radiology, Mie University Graduate School of Medicine, Tsu, Mie, Japan; Department of Cardiology and Nephrology, Mie University Graduate School of Medicine, 2-174 Edobashi, Tsu, Mie 5148507, Japan

**Keywords:** Cardio-oncology, Takotsubo syndrome, Cancer therapy, Immune checkpoint inhibitors, Endomyocardial biopsy, Case report

## Abstract

**Background:**

Takotsubo syndrome (TTS) is characterized by transient regional left ventricular (LV) dysfunction occurring in individuals exposed to physical or emotional stress. Various stressors are triggers for TTS in cancer patients, and anti-cancer drugs have recently been proposed as a trigger. Therefore, further studies are needed to clarify these triggers and avoid the unnecessary interruption of anti-cancer treatment.

**Case summary:**

A 66-year-old woman presented with dyspnoea 10 days after the initiation of atezolizumab in combination with bevacizumab. She had previously received osimertinib as first-line therapy for recurrent lung cancer after primary resection and atezolizumab in combination with bevacizumab, paclitaxel, and carboplatin as second-line therapy. She was admitted due to electrocardiography abnormalities and elevated troponin I and brain natriuretic peptide levels. Echocardiography revealed circumferential severe LV hypokinesis at the mid-ventricular level, with preserved wall motion at the base and apex. Cardiac catheterization performed after the attenuation of symptoms with 20 mg of intravenous furosemide showed normal coronary arteries. Cardiac magnetic resonance imaging on Day 4 revealed increases in T_1_ and T_2_ values and extracellular volume fraction; however, neither myocardial infiltration of inflammatory cells or myocardial necrosis was observed in endomyocardial samples obtained on the day of her arrival. Atypical TTS was suspected, and she was treated with perindopril, bisoprolol, and spironolactone. Magnetic resonance imaging 1.5 months after the onset of TTS showed improvements in LV contractility, T_1_ and T_2_ values, and the extracellular volume fraction.

**Discussion:**

A more detailed understanding of the relationship between anti-cancer drugs and TTS is crucial for preventing interruptions to anti-cancer therapy.

**Learning points**

- To understand the importance of establishing an accurate differential diagnosis between the potential causes of new acute cardiac dysfunction with abnormal segmental wall motion in patients with active cancer.
- To understand the importance of myocardial biopsy in patients with cardiac events under immune checkpoint inhibitor treatment.

## Introduction

Takotsubo syndrome (TTS) is a syndrome characterized by transient regional dysfunction of the left ventricle occurring in individuals exposed to physical or emotional stress. Cancer patients have a higher incidence of TTS than those without cancer, with a higher rate of mortality.^1–4^ They have multiple potential stressors mainly due to the mental strain caused by the diagnosis of cancer, the symptoms of cancer, and the physical strain of invasive treatment.^1^ Anti-cancer drugs have recently been proposed as a risk factor for its development.^1^ Therefore, further studies are needed to clarify these triggers and avoid the unnecessary interruption of anti-cancer treatment.

## Summary figure

**Table:** 

Timeline	Previous history of anti-cancer therapy with osimertinib
In the past 5 months	4 courses of atezolizumab in combination with bevacizumab, paclitaxel, and carboplatin.
10 days before admission	First cycle of maintenance therapy with a combination of atezolizumab and bevacizumab.
4 days before admission	Emotional stress due to domestic issues.
3 days before admission	She noted progressive dyspnoea.
On admission (Day 0)	She presented with hypoxia and leg oedema. Electrocardiogram showed poor R-wave progression in precordial leads and negative T-waves in leads Ⅱ, Ⅲ, and aVF. Elevated brain natriuretic peptide (BNP) and troponin I (TnI) levels were observed. Coronary angiography showed normal coronary arteries. Treatment with intravenous furosemide. The initiation of oral perindopril and bisoprolol.
Day 2	Decreases in BNP and TnI levels.
Day 4	Cardiac magnetic resonance imaging showed circumferential severe left ventricular (LV) hypokinesis at the mid-ventricular level and an increased LV mass. Native T_1_, extracellular volume fraction (ECV), and global T_2_ were elevated. Myocardial biopsy samples showed a small number of inflammatory cells and interstitial oedema without myocardial necrosis.
Day 10	She was discharged.
Day 78	First initiation of third-line therapy with docetaxel.
Day 103	She presented with elevated TnI and BNP levels. Echocardiography and cardiac magnetic resonance (CMR) revealed mid-ventricular hypokinesis. Diagnosis of recurrent Takotsubo syndrome and heart failure therapy was titrated.
Day 133	Recovery of LV wall motion.

## Case presentation

A 66-year-old woman with recurrent lung cancer after primary tumour resection had been receiving molecular target therapy with osimertinib for 2 years and 9 months. As the cancer progressed, she completed four courses of atezolizumab in combination with bevacizumab, paclitaxel, and carboplatin as second-line therapy. She was receiving maintenance therapy with a combination of atezolizumab and bevacizumab. Electrocardiography performed at the baseline and at each interval of second-line therapy was normal with no ST-T changes. She had continuous strong malaise from the last combination therapy and experienced emotional stress due to domestic issues 4 days before arrival and noted progressive dyspnoea from the next day. Her symptoms began with shortness of breath on exertion and progressed to orthopnoea at the time of presentation.

On admission, blood pressure was 102/62 mmHg; heart rate, 98 b.p.m.; respiratory rate, 22 breaths/min; and oxygen saturation, 96% with 1 L/min via a nasal cannula. A physical examination revealed slight leg oedema, but no jugular venous distention or abnormalities in cardiac sounds. Pulmonary congestion and cardiac enlargement with a cardiothoracic ratio of 58% were noted on chest X-ray. Electrocardiography showed the poor progression of R-waves in the precordial leads and negative T-waves in leads Ⅱ, Ⅲ, and aVF (see Supplementary material online, *Figure S1*). Laboratory test results on admission were as follows: creatinine kinase, 164 U/L (normal range, 59–248 U/L); BNP, 1305.6 pg/mL (normal range, <18.4 pg/mL); TnI, 284.5 pg/mL (normal range, <34.2 pg/mL); C-reactive protein, 2.4 mg/dL; urea nitrogen, 24.0 mg/dL; creatinine, 0.91 mg/dL; eGFR, 47.8 mL/min/1.73 m^2^; total bilirubin, 0.6 mg/dL; AST, 30 U/L; ALT, 26 U/L; LDH, 252 U/L; and lactate, 1.1 mmol/L. Echocardiography revealed a reduced LV ejection fraction (LVEF) of 35%, circumferential severe hypokinesis of the mid-ventricular level, and preserved wall motion at other sections of the LV, including the LV apex. No pericardial effusion or thickening of the LV wall was observed. She was treated with 20 mg of intravenous furosemide, which resulted in a large amount of diuresis and the rapid attenuation of symptoms. The patient underwent cardiac catheterization on the day of her admission. Coronary angiography showed normal coronary arteries, and right heart catheterization revealed a cardiac index of 2.64 L/min/m^2^ and normal pulmonary capillary wedge pressure of 9 mmHg. Left ventriculography was not performed. Steroids were not administered due to the lack of active symptoms for myocarditis, such as myocardial oedema, obvious pericardial effusion, ventricular arrhythmia, and an atrioventricular block. Moreover, due to the unique wall motion abnormality and mental stress just before the onset of symptoms, we assumed that TSS was more likely.

**Figure 1:**
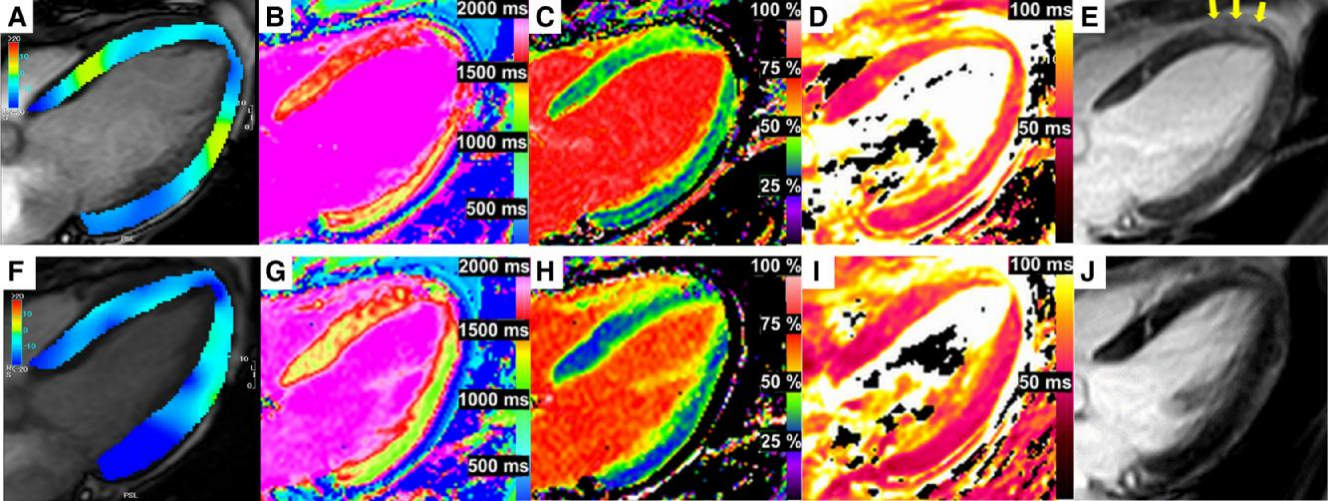
Cardiac magnetic resonance abnormalities and recovery. Cardiac magnetic resonance imaging on Day 4 (*A–E*) and Day 59 (*F–J*). A coloured strain analysis (Circle CVI42®) with left ventricular mid-ventricular hypokinesis (*A*) and recovered wall motion (*F*). Increased native T_1_ (*B*), extracellular volume fraction (*C*), and T_2_ (*D*) on Day 4 and normalized native T_1_ (*G*), extracellular volume fraction (*H*), and T_2_ (*I*) on Day 59. Focal late gadolinium enhancement (arrow) at baseline (*E*) and its disappearance in the follow-up (*J*).

**Figure 2:**
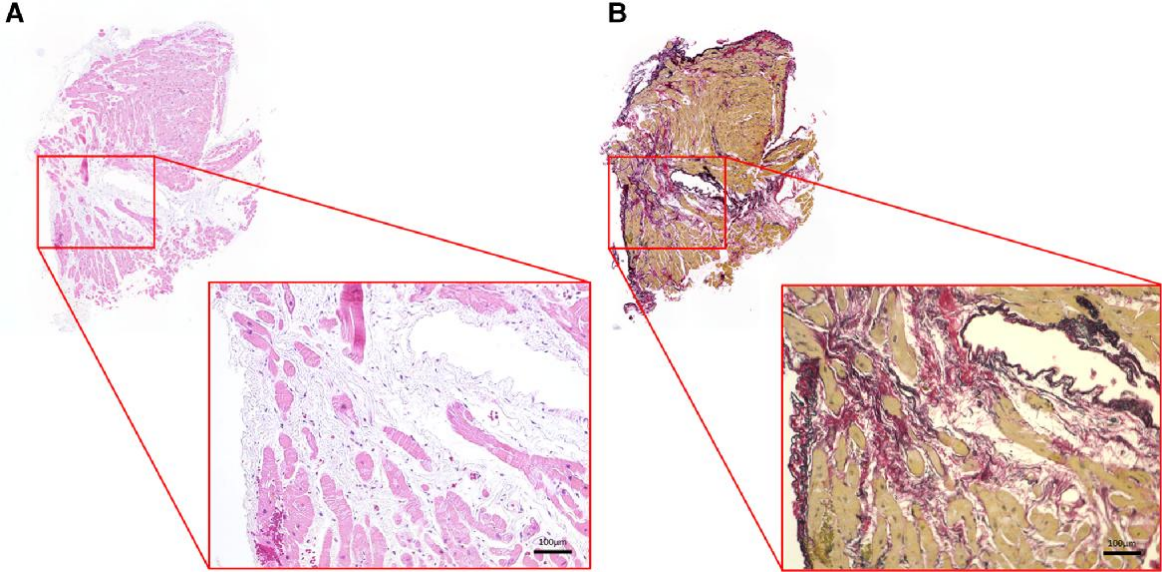
Histological analysis of the myocardium. (*A*) Haematoxylin–eosin staining showing a small amount of inflammatory cell infiltration and interstitial oedema. (*B*) Elastica–Picrosirius red staining showing a small area of fibrosis.

Cardiac magnetic resonance on Day 4 demonstrated reduced LVEF of 34% with circumferential severe LV hypokinesis of the mid-ventricular level by analysing global longitudinal strain with feature tracking method (longitudinal peak strain, base −10.8%, mid −5.4%, and apex −8.8%) (*Figure 1A*), increased LV mass index of 76.0 g/m^2^, preserved right ventricular ejection fraction of 49%, and slight pericardial effusion. Multi-parametric mapping revealed prolonged global native T_1_ (1447 ms; normal range, 1294 ± 39 ms) and an increased ECV (37%; normal range, 26.1 ± 1.4%) predominantly at the septum (*Figure 1B* and *C*). A diffuse high signal intensity on T_2_-weighted short Tl inversion recovery (not shown) and prolonged global T_2_ (57.6 ms; normal range, 45 ± 5 ms) on the T_2_ map (*Figure 1D*) suggested acute diffuse myocardial oedema. Faint enhancement observed at the septum on late gadolinium enhancement (LGE, *Figure 1E*) was considered to reflect myocardial oedema. High signals at the basal septum in *Figure 1E* and *F* were confirmed to be LV myocardial crypts based on cine images. Endomyocardial biopsy from the mid-right interventricular septum was performed on admission, and three samples were obtained. Biopsy samples showed a small number of inflammatory cells and interstitial oedema without myocardial necrosis (*Figure 2A*); therefore, myocarditis was considered to be histologically unlikely. Elastica–Picrosirius red staining also revealed small replacement fibrosis indicating previous myocardial injury (*Figure 2B*). These imaging results and cardiac wall motion characteristics suggested acute TTS coupled with pre-existing myocardial injury.

**Figure 3:**
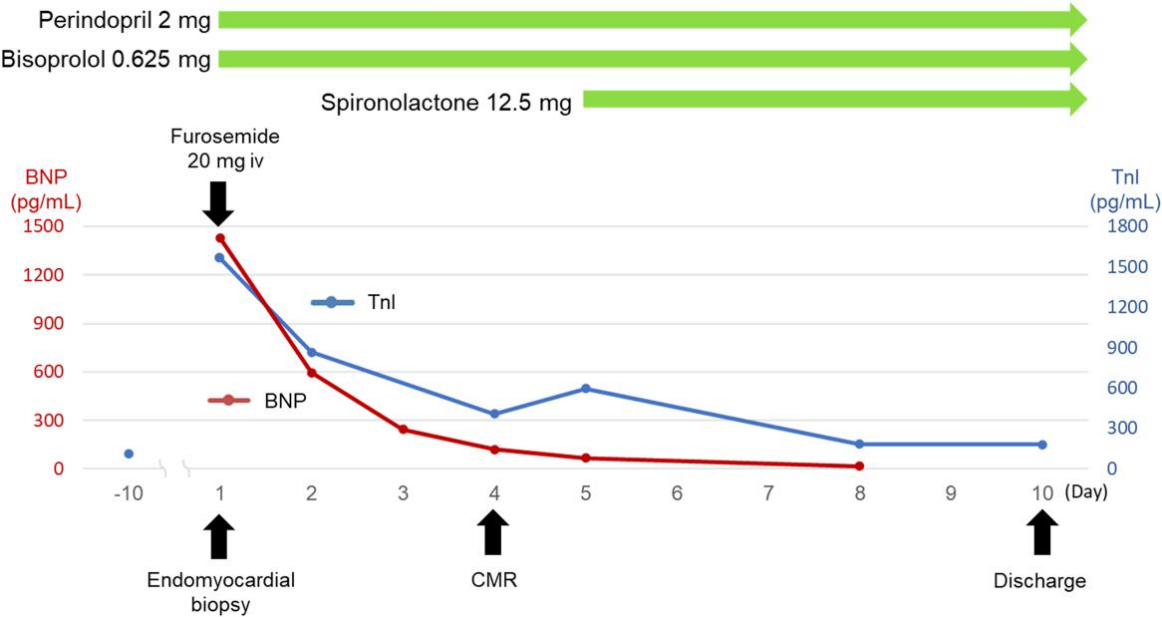
Timeline of biomarkers and treatment approach.

Treatment with oral perindopril (2 mg/day), which was half the initial recommended dose in Japan, bisoprolol (0.625 mg/day), and spironolactone (12.5 mg/day) was initiated. The patient was asymptomatic on the fourth hospital day and was discharged on Day 10 (*Figure 3*). Brain natriuretic peptide and TnI levels at discharge were 65.5 and <10.0 pg/mL, respectively. Since tumour progression was suspected based on elevated tumour markers, a third course of atezolizumab and bevacizumab was not administered. Cardiac magnetic resonance performed on Day 59 confirmed the significant recovery of mid-LV akinesis and an improvement in LVEF to ∼50% accompanied by the recovery of native T_1_, ECV, and T_2_ values to within the normal range (*Figure 1G–I*). The patient was diagnosed with TTS according to the 2004 Mayo Clinic diagnostic criteria.^5^ On Day 81, third-line therapy with docetaxel and ramucirumab was initiated. She presented on Day 103 with orthopnoea and leg oedema as well as elevated TnI and BNP levels. Electrocardiography revealed negative T-waves in the same leads as those in the first event. Echocardiography and CMR showed decreased LVEF (38% by CMR) with severe hypokinesis localized at the mid-ventricular level (not shown). The patient was diagnosed with the recurrence of TTS, and docetaxel was discontinued. Perindopril and bisoprolol were up-titrated to 4 and 3.125 mg/day, respectively. Left ventricular wall motion had recovered by the 1-month follow-up (Supplementary Videos S1–S4).

## Discussion

A number of anti-cancer agents have been reported as a possible cause of TTS. 5-Fluorouracil and capecitabine are the most commonly reported anti-cancer agents as a trigger of TTS. In addition, all the agents recently administered in this case have been proposed as triggers of TTS.^1,2,6^ The present case highlights the challenges associated with identifying the triggers of TTS in cancer patients, particularly when patients have multiple stressors and are receiving anti-cancer treatments. Our patient developed TTS after 1 week of atezolizumab in combination with bevacizumab following 4 months of atezolizumab in combination with bevacizumab, paclitaxel, and carboplatin. The majority of previously reported cases of TTS were associated with the first cycle of anti-cancer therapy.^2,6^ Although drug-induced TTS is a possible explanation in the present case, we assumed that the strong physical and emotional stresses experienced by the patient just before the onset of TTS were the trigger. However, she experienced a second event of TTS without any emotional stress, but with the use of docetaxel, which is a potential trigger for TTS. Therefore, it is important to note that cancer patients may develop TSS more than once with different triggers.^2^

The present case also highlights the pitfalls of diagnosing the phenotype of cardiotoxicity during immune checkpoint inhibitor (ICI) therapy. Immune checkpoint inhibitors are increasingly used for various cancer patients as an effective treatment for advanced cancers; however, blocking the immune brake may result in autoimmune reactions as a side effect. Myocarditis is the most common cardiovascular immune-related adverse event and requires the early initiation of steroid therapy due to its high mortality rate. Therefore, myocarditis is the most important and widely known differential diagnosis of myocardial injury during ICI therapy.^7^ However, it is necessary to consider the possibility of other cardiovascular events, such as TTS, non-inflammatory LV dysfunction, pericarditis, and arrhythmia, because of the different therapeutic approaches required. In some TTS cases, it is difficult to differentiate TTS from myocarditis because of the overlapping clinical and imaging features.^8^ In the present case, the patient had successfully completed the first 12 weeks of treatment, a period during which myocarditis is the most likely to develop. The level of TnI spontaneously decreased on the following day. Furthermore, there was no ventricular arrhythmia or haemodynamic instability, both of which are high-risk factors for myocarditis. These findings allowed us to avoid unnecessary steroid therapy. Steroids are recommended if any of the above is detected.

Cardiac magnetic resonance is the recommended non-invasive tool to diagnose myocarditis based on the 2018 Lake Louise criteria.^9^ These criteria require both myocardial oedema and non-ischaemic myocardial injury, which is confirmed by increases in native T_1_, ECV, the regional LGE signal, native T_2_, and T_2_ signal intensity. Since these features are found in patients with an inflammatory-related state, myocarditis may be difficult to distinguish from TTS. In the present case, a histological analysis allowed us to exclude myocarditis and we treated the patient with supportive heart failure therapy and did not administer steroid therapy.

## Conclusion

We herein report a case of acute myocardial injury during the administration of a combination of cytotoxic chemotherapy, molecular targeted therapy, and immunotherapy with the co-existence of emotional and physical stresses. A histological analysis is useful for differentiating myocarditis from TTS in the setting of overlapping clinical features and CMR findings. Since the interruption of anti-cancer therapy affects patient outcomes, a more detailed understanding of the relationship between anti-cancer drugs and TTS is crucial for the selection of therapeutic strategies, including the interruption of anti-cancer therapy.

## Lead author biography


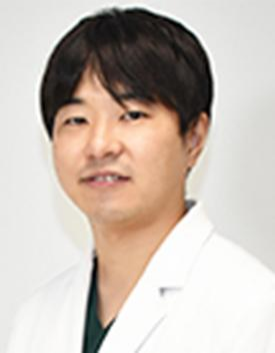


Keita Yamada is a cardiologist at Mie University Hospital, Mie, Japan. He is currently training in clinical cardiology, and he is interested in the field of heart failure.

## Supplementary Material

ytae355_Supplementary_Data

## Data Availability

The authors confirm that the data supporting the findings of this case report are available within the manuscript and its online Supplementary material. Further details can be requested by contacting the corresponding author.

## Supplementary material

**Consent:** The authors confirm that written consent for submission and publication of this case report, including images and associated text, has been obtained from the patient in line with COPE guidance.

**Funding:** None declared.
