# Supplementary material for: Takotsubo syndrome in a cancer patient treated with a combination of anti-cancer drugs including immune checkpoint inhibitors: a case report
Source: Eur Heart J Case Rep. 2024 Jul 23;8(8):ytae355. doi: 10.1093/ehjcr/ytae355 (PMC11299031; doi:10.1093/ehjcr/ytae355)
Supplement: ytae355_Supplementary_Data [file ytae355_supplementary_data.zip › supplementary Figure 1.pptx]

## Slide 1
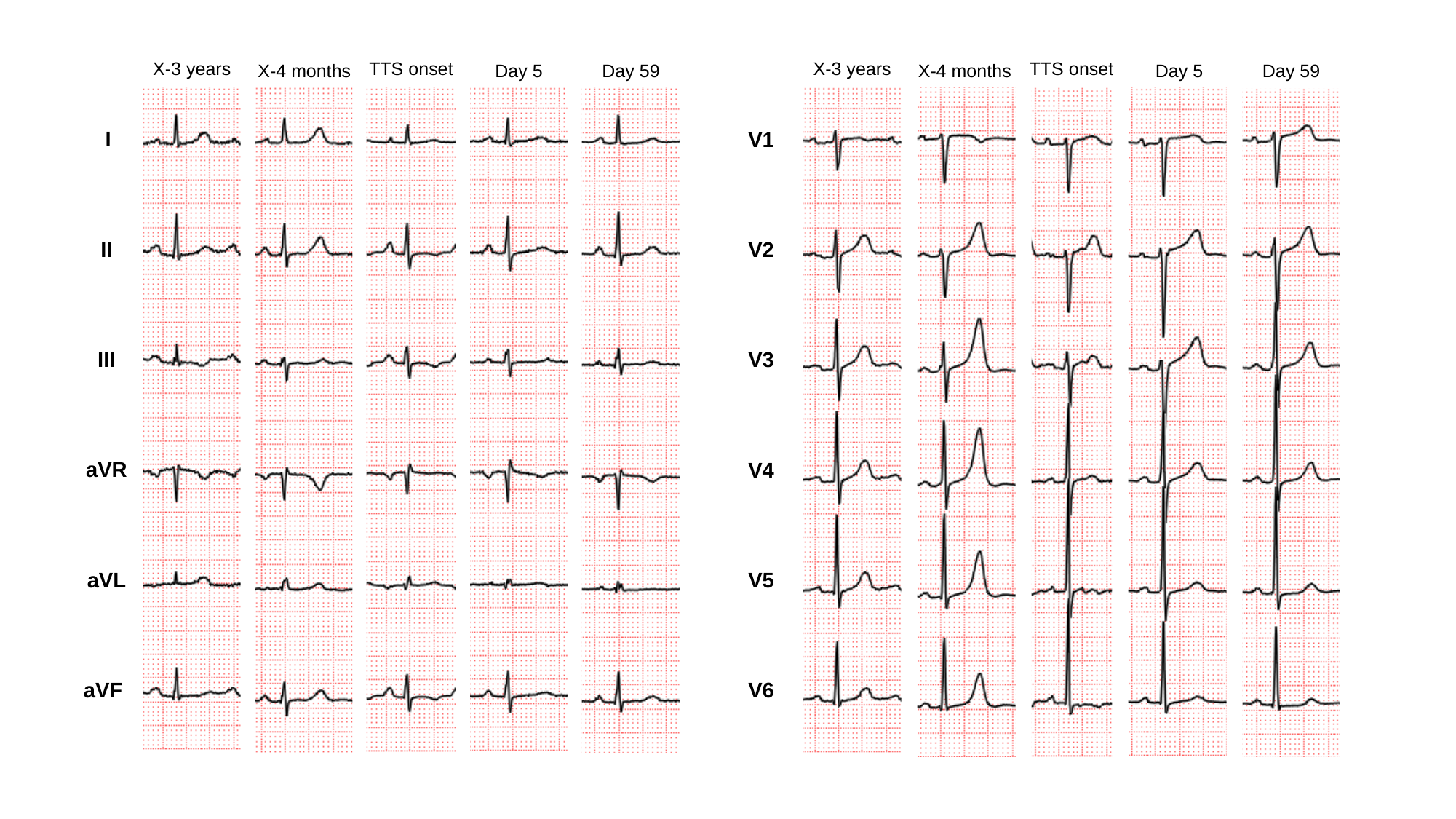

TTS onset
TTS onset
X-3 years
X-3 years
X-4 months
Day 5
Day 59
X-4 months
Day 5
Day 59
I
V1
II
V2
III
V3
aVR
V4
aVL
V5
V6
aVF
